# Laparoscopic Versus Open Approach for Strangulated Small Bowel Obstruction: A Propensity Score–Matched Analysis

**DOI:** 10.1002/wjs.70253

**Published:** 2026-02-03

**Authors:** Toshimichi Kobayashi, Ryota Suda, Hiroki Yamaguchi, Shoma Iida, Kanami Iwama, Takumi Seichi, Yoshihiro Nagae, Hiroyuki Higuchi, Akitoshi Ando, Itsuki Koganezawa, Masashi Nakagawa, Kei Yokozuka, Shigeto Ochiai, Takahiro Gunji, Toru Sano, Satoshi Tabuchi, Naokazu Chiba, Shigeyuki Kawachi

**Affiliations:** ^1^ Department of Digestive and Transplantation Surgery Tokyo Medical University Hachioji Medical Center Tokyo Japan

**Keywords:** conversion to open surgery, laparoscopic surgery, propensity score matching, strangulated small bowel obstruction

## Abstract

**Background:**

Laparoscopic surgery has gradually gained acceptance for abdominal surgical emergencies; however, limited reports exist on laparoscopic surgery for strangulated small bowel obstruction (SSBO). We aimed to demonstrate the efficacy and feasibility of laparoscopic surgery for SSBO.

**Methods:**

In this single‐center retrospective study, patients who underwent emergency surgery for SSBO between January 2014 and December 2024 were included and divided into laparoscopy and open groups. Propensity score matching (PSM) was performed to compare the primary outcomes—intraoperative and short‐term postoperative outcomes—between the groups. Logistic regression analysis was used to identify the factors associated with the conversion from laparoscopic to open surgery as secondary outcomes.

**Results:**

A total of 123 patients were included in this study, of whom 39 (31.7%) were assigned to the laparoscopy group. After PSM, the overall rate of the Clavien–Dindo grade ≥ II postoperative complications was significantly lower in the laparoscopy than the open group (7.4% vs. 29.6%; *p* = 0.036). Of the 39 patients in the laparoscopy group, 10 (25.6%) were converted from laparoscopic to open surgery. The number of previous laparotomies (odds ratio: 4.036, 95% confidence interval: 1.189–13.701, and *p* = 0.025) and a history of gastrointestinal surgery (odds ratio: 6.125, 95% confidence interval: 1.263–29.699, and *p* = 0.024) were identified as factors significantly associated with conversion from laparoscopic to open surgery in patients with SSBO.

**Conclusion:**

Our study suggests that laparoscopic surgery for SSBO is beneficial for reducing the occurrence of postoperative complications. However, laparoscopic surgery should be performed in patients with a history of multiple laparotomies or gastrointestinal surgery, considering the possibility of conversion to open surgery.

## Graphical abstract

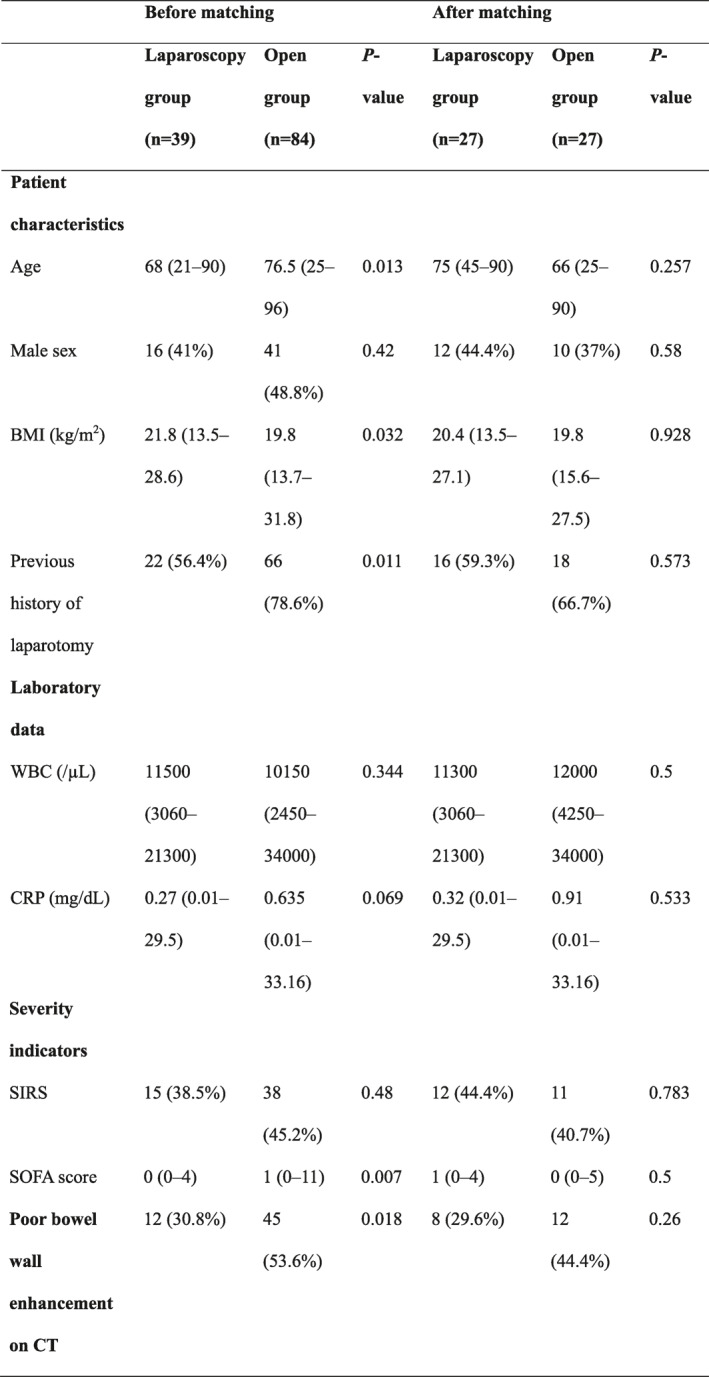

## 1 Introduction

Strangulated small bowel obstruction (SSBO) is an abdominal surgical emergency that leads to intestinal ischemia, necrosis, and eventually perforation due to impaired blood flow caused by compression of the mesenteric vessels [1, 2]. SSBO with intestinal necrosis is reported to have a mortality of 16%–40%, and early diagnosis and intervention play significant roles in the improvement of outcomes [3, 4, 5].

Laparoscopic surgery is a well‐established surgical procedure for elective abdominal surgery. It has gradually become an accepted approach for abdominal surgical emergencies such as acute appendicitis, acute cholecystitis, and perforated peptic ulcer [6]. Adhesive small bowel obstruction, a common postoperative complication after abdominal surgery, requires operative management in 20%–30% of cases [7]. Several previous systematic reviews and meta‐analyses have reported the efficacy of laparoscopic surgery compared with open surgery for adhesive small bowel obstruction [8, 9, 10, 11]. SSBO is defined as small bowel obstruction with intestinal ischemia due to strangulation of mesenteric vessels and occurs in approximately 10% of cases of small bowel obstruction [12]; however, few studies have focused on the outcomes of laparoscopic surgery for SSBO. We aimed to demonstrate the efficacy and feasibility of laparoscopic surgery for treating SSBO.

## 2 Methods

### 2.1 Study Design and Population

This single‐center retrospective study was conducted at Tokyo Medical University Hachioji Medical Center. Patients who underwent emergency surgery for SSBO at our institution between January 2014 and December 2024 were included. Electronic medical records were reviewed, and patient characteristics and perioperative data were extracted. SSBO was diagnosed based on medical history and computed tomography (CT) imaging findings and was definitively confirmed by intraoperative findings. Patients with groin or ventral hernias were excluded. Finally, we divided the enrolled patients into laparoscopic approach (laparoscopy) group and open approach (open) groups. Patients who required conversion from laparoscopic surgery to open surgery were assigned to the laparoscopy group; a small incision for the resection of the intestine and assessment of the viability of the ischemic intestine was not considered a conversion to open surgery. In this study, the laparoscopic approach was defined as rocedures in which the primary procedure, including the release of strangulation, is completed laparoscopically, even if a small auxiliary incision (≤ 7 cm) is required for bowel assessment or specimen extraction.

### 2.2 Surgical Approach and Procedures

Patients with preoperative hemodynamic instability or bowel perforation on CT underwent an open approach, whereas patients without these findings underwent either an open or a laparoscopic approach at the discretion of the surgeon. Laparoscopic surgery was performed using a three‐port technique with a 12‐mm camera port and two 5‐ or 12‐mm ports; additional ports were inserted as required. A 12‐mm camera port was inserted into a site that appeared free of adhesions on CT imaging, and other ports were inserted into sites free of adhesions under laparoscopic vision. After the strangulation was laparoscopically released, the degree of ischemic change in the strangulated intestine was evaluated by the surgeons. When irreversible ischemic changes were laparoscopically suspected, a small longitudinal incision (≤ 7 cm) was made at the umbilicus; the need for bowel resection was determined under direct observation. Conversion from laparoscopic to open surgery was performed when it was difficult to release the strangulation laparoscopically owing to the inability to secure the surgical field of view or the risk of iatrogenic bowel injury due to the dissection of adhesions.

### 2.3 Data Collection

Demographic characteristics of the study population and preoperative variables included age, sex, body mass index (BMI), previous history of laparotomy, laboratory data (white blood cell [WBC], C‐reactive protein [CRP], hemoglobin, platelet, albumin, bilirubin, and creatinine), CT findings (peritoneal fluid, poor bowel wall enhancement, and CT value of peritoneal fluid), and severity indicators (systemic inflammatory response syndrome [SIRS] and sequential organ failure assessment [SOFA] score). Laboratory data and severity indicators were defined as those evaluated immediately before surgery. Perioperative variables included bowel resection, conversion to open surgery, operation time, blood loss, interval from operation to oral feeding, postoperative complications, length of postoperative hospital stay, and mortality.

### 2.4 Statistical Analyses

The primary outcomes were intraoperative and short‐term postoperative outcomes. Demographic characteristics and clinical variables were compared between the laparoscopy and open groups. Propensity score matching (PSM) was performed to minimize the inherent selection bias of retrospective observational studies. The propensity score was estimated using the logistic regression model with the following variables: age, sex, BMI, previous history of laparotomy, WBC, CRP, SIRS, SOFA score, and poor bowel wall enhancement on CT. Patients in each group were matched using one‐to‐one nearest‐neighbor matching without replacement, with a caliper of 0.2 standard deviations of the logit of the estimated propensity score. Continuous variables were expressed as medians with ranges, and categorical variables were expressed as frequencies with percentages. The Mann–Whitney *U* test was used to compare continuous variables, and the chi‐square test or Fisher's exact test was used to compare categorical variables.

The secondary outcome was the identification of factors associated with conversion from laparoscopic to open surgery via logistic regression analysis. All statistical analyses were performed using SPSS Statistics (Version 29; IBM Corp., Armonk, NY, USA). Statistical significance was set at *p* < 0.05.

## 3 Results

### 3.1 Patient Characteristics

During the inclusion period, 123 patients underwent emergency surgery with a diagnosis of SSBO. Of these, 39 and 84 patients were assigned to the laparoscopy and open groups, respectively. PSM yielded 27 patients in each group. Patient characteristics of the two groups are shown in Table 1.

**TABLE 1:** Demographic and clinical characteristics.

	Before matching			After matching		
	Laparoscopy group (*n* = 39)	Open group (*n* = 84)	*p* value	Laparoscopy group (*n* = 27)	Open group (*n* = 27)	*p* value
Patient characteristics						
Age	68 (21–90)	76.5 (25–96)	0.013	75 (45–90)	66 (25–90)	0.257
Male sex	16 (41%)	41 (48.8%)	0.42	12 (44.4%)	10 (37%)	0.58
BMI (kg/m^2^)	21.8 (13.5–28.6)	19.8 (13.7–31.8)	0.032	20.4 (13.5–27.1)	19.8 (15.6–27.5)	0.928
Previous history of laparotomy	22 (56.4%)	66 (78.6%)	0.011	16 (59.3%)	18 (66.7%)	0.573
Laboratory data						
WBC (/µL)	11,500 (3060–21300)	10,150 (2450–34000)	0.344	11,300 (3060–21300)	12,000 (4250–34000)	0.5
CRP (mg/dL)	0.27 (0.01–29.5)	0.635 (0.01–33.16)	0.069	0.32 (0.01–29.5)	0.91 (0.01–33.16)	0.533
Severity indicators						
SIRS	15 (38.5%)	38 (45.2%)	0.48	12 (44.4%)	11 (40.7%)	0.783
SOFA score	0 (0–4)	1 (0–11)	0.007	1 (0–4)	0 (0–5)	0.5
Poor bowel wall enhancement on CT	12 (30.8%)	45 (53.6%)	0.018	8 (29.6%)	12 (44.4%)	0.26

*Note:* Categorical data are expressed as percentages, and continuous data are expressed as median (min–max).

Abbreviations: BMI, body mass index; CRP, C‐reactive protein; CT, computed tomography; SIRS, systemic inflammatory response syndrome; SOFA, sequential organ failure assessment; WBC, white blood cell.

Before matching, patients in the laparoscopy group were significantly younger (*p* = 0.013) and had a significantly higher BMI (*p* = 0.032), fewer previous laparotomies (*p* = 0.011), lower SOFA scores (*p* = 0.007), and less frequent poor bowel wall enhancement on CT (*p* = 0.018) than those in the open group. No significant differences were observed among laboratory data; however, CRP levels tended to be lower in the laparoscopy group than in the open group (*p* = 0.069). After matching, all variables were well balanced, and no significant differences were observed between the two groups regarding any of the clinical variables.

### 3.2 Comparison of Intraoperative and Short‐Term Postoperative Outcomes After PSM

The results are summarized in Table 2. The overall rate of the Clavien–Dindo grade ≥ II postoperative complications was significantly lower in the laparoscopy than in the open group (7.4% vs. 29.6% and *p* = 0.036). No significant difference was observed regarding the rate of complications of the Clavien–Dindo grade ≥ III (7.4% vs. 14.8% and *p* = 0.334). As the number of these individual complications was small, statistical comparisons were not performed; a descriptive list of all postoperative complications with their corresponding Clavien–Dindo grades is provided in Table 3. Several clinically significant complications (Clavien–Dindo grade II–III) were observed in the open group, including grade III surgical site infection (*n* = 2), grade II–III ileus (*n* = 3), and grade II–III pneumonia (*n* = 2). By contrast, these higher grade complications were either absent or rare in the laparoscopy group. No significant differences were observed regarding operation time, blood loss, interval from operation to oral feeding, postoperative hospital stay, or mortality.

**TABLE 2:** Comparison of intraoperative and short‐term postoperative outcomes after propensity score matching.

	Laparoscopy group (*n* = 27)	Open group (*n* = 27)	*p* value
Bowel resection	10 (37%)	14 (51.9%)	0.273
Operation time (min)	84 (36–168)	90 (46–230)	0.243
Blood loss (mL)	10 (5–1570)	50 (5–1300)	0.111
Interval from operation to oral feeding (day)	5 (2–8)	5 (2–12)	0.176
Postoperative complications CD grade ≥ II	2 (7.4%)	8 (29.6%)	0.036
Postoperative complications CD grade ≥ III	2 (7.4%)	4 (14.8%)	0.334
Postoperative hospital stay (day)	11 (5–24)	13 (5–40)	0.248
Mortality	0 (0%)	0 (0%)	—

*Note:* Categorical data are expressed as percentages, and continuous data are expressed as median (min–max).

Abbreviation: CD, Clavien–Dindo.

**TABLE 3:** Postoperative complications after propensity score matching with corresponding CD grades.

Complication	CD grade	Laparoscopy group (*n* = 27) (%)	Open group (*n* = 27) (%)
Surgical site infection	I	2 (5.4)	1 (2.7)
	II	0 (0)	1 (2.7)
	III	0 (0)	2 (5.4)
Ileus	I	1 (2.7)	0 (0)
	II	0 (0)	2 (5.4)
	III	0 (0)	1 (2.7)
Pneumonia	II	0 (0)	1 (2.7)
	III	0 (0)	1 (2.7)
Anastomotic bleeding	III	1 (2.7)	0 (0)
Pleural effusion	III	1 (2.7)	0 (0)

*Note:* Categorical data are expressed as percentages.

Abbreviation: CD, Clavien–Dindo.

### 3.3 Factors Associated With Conversion From Laparoscopic to Open Surgery

Of the 39 patients in the laparoscopy group, 10 (25.6%) were converted from laparoscopic to open surgery. The reasons for conversion were categorized into clinically meaningful groups as follows: inability to release strangulation due to complex adhesions (*n* = 4), massive adhesions (*n* = 3), poor surgical field of view (*n* = 2), and extensive irreversible intestinal ischemia (*n* = 1). No conversions were performed due to intraoperative bowel injury. The results of the logistic regression analysis of the factors associated with conversion to open surgery are shown in Table 4. Given the small number of conversion events, multivariate logistic regression analysis was not performed. In the univariate logistic regression analysis, the number of previous laparotomies (odds ratio: 4.036 with 95% confidence interval: 1.189–13.701; *p* = 0.025) and a history of gastrointestinal surgery (odds ratio: 6.125 with 95% confidence interval: 1.263–29.699; *p* = 0.024) were identified as factors significantly associated with conversion from laparoscopic to open surgery.

**TABLE 4:** Factors associated with conversion from laparoscopic to open surgery.

	Univariate logistic regression analysis		
	OR	95% CI	*p* value
Patient characteristics			
Age ≥ 75 (years)	1.417	0.335–5.998	0.636
Male sex	0.611	0.144–2.602	0.505
BMI ≥ 25 (kg/m^2^)	0	0	0.999
Previous history of laparotomy	4.286	0.773–23.746	0.096
Number of previous laparotomies	4.036	1.189–13.701	0.025
History of gastrointestinal surgery	6.125	1.263–29.699	0.024
History of obstetrical and gynecological surgery	0.958	0.160–5.746	0.963
Laboratory data			
WBC (×10^3^/µL)	1.045	0.874–1.249	0.63
CRP (mg/dL)	0.981	0.872–1.105	0.755
Hb (g/dL)	0.814	0.587–1.13	0.218
Plt (×10^4^/μL)	1.02	0.924–1.125	0.7
Alb (g/dL)	1.002	0.354–2.83	0.998
Bil (mg/dL)	0.235	0.024–2.338	0.216
Cre (mg/dL)	1.821	0.355–9.347	0.473
CT findings			
Peritoneal fluid	2.348	0.247–22.34	0.458
Poor bowel wall enhancement	0.389	0.041–3.713	0.412
CT value of peritoneal fluid (HU)	1.025	0.928–1.131	0.632
Severity indicators			
SIRS	0.607	0.13–2.836	0.526
SOFA score	0.88	0.421–1.839	0.733
Bowel resection	2.222	0.512–9.647	0.286

Abbreviations: Alb, albumin; Bil, bilirubin; BMI, body mass index; CI, confidence interval; Cre, creatinine; CRP, C‐reactive protein; Hb, hemoglobin; HU, Hounsfield unit; OR, odds ratio; Plt, platelet; SIRS, systemic inflammatory response syndrome; SOFA, sequential organ failure assessment; WBC, white blood cell.

## 4 Discussion

Our study compared the intraoperative and short‐term postoperative outcomes of laparoscopic versus open surgery for SSBO. PSM analysis showed that the occurrence of postoperative complications (Clavien–Dindo grade ≥ II) was significantly lower in the laparoscopy group than in the open group. Additionally, the number of previous laparotomies and a history of gastrointestinal surgery were identified as factors significantly associated with conversion from laparoscopic to open surgery. To our knowledge, this is the first study to examine the efficacy and feasibility of laparoscopic surgery for SSBO using PSM. Currently, there is no universally standardized treatment protocol for the laparoscopic management of SSBO, and treatment strategies may vary substantially among institutions. In this context, the present study provides real‐world data that may help inform clinical decision‐making in this relatively understudied field.

Laparoscopic surgery has become the standard surgical approach for elective surgery in various fields, as well as for many abdominal surgical emergencies. In the Cesena guidelines published by the World Society of Emergency Surgery, the laparoscopic approach is suggested to be a safe, feasible, and effective therapeutic option for hemodynamically stable patients undergoing emergency abdominal surgery for general surgery emergencies and abdominal trauma [6]. In previous systematic reviews and meta‐analyses on adhesive small bowel obstruction, laparoscopic surgery was reported to be associated with a decrease in postoperative complications, length of hospital stay, and mortality when compared with open surgery [8, 9, 10, 11]. In an international, multicenter, randomized, and open‐label trial (LASSO trial), laparoscopic surgery for adhesive small bowel obstruction was reported to provide quicker recovery and return of bowel function in selected patients than open surgery [13]. By contrast, only one study focused on SSBO, comparing the outcomes of laparoscopic and open surgery. In a retrospective cohort study without PSM, Kohga et al. [14] reported that laparoscopic surgery for patients with SSBO who did not require bowel resection yielded superior outcomes, especially regarding the time from surgery to ingestion and postoperative use of analgesic agents. In contrast to patients with adhesive small bowel obstruction without intestinal ischemia, patients with SSBO are at risk of bowel resection and declining conditions due to progressive intestinal ischemia. Thus, we applied PSM to optimize the balance of covariates, including inflammatory markers and severity indicators, to reduce confounding between the two surgical approaches. After matching, the rate of overall postoperative complications (Clavien–Dindo grade ≥ II) was significantly lower in the laparoscopy than the open group. Additionally, the rates of surgical site infection (Clavien–Dindo grade ≥ II) and ileus (Clavien–Dindo grade ≥ II) were lower in the laparoscopy than the open group, although with no statistically significant difference; these results were consistent with those of previous studies on adhesive small bowel obstruction [8, 9, 10, 11, 13]. These results can be attributed to the smaller incisions and reduced intestinal irritation associated with the laparoscopic approach, even when bowel resection is required, as reported in previous studies [9, 14].

Although laparoscopic surgery offers several advantages as a minimally invasive procedure, it has the limitation of being converted to open surgery. In our study, the rate of conversion from laparoscopic surgery to open surgery was 25.6%, consistent with the results of previous studies [13, 14]. This conversion rate is higher than that of recent studies on surgical abdominal emergencies mainly involving inflammation, such as appendicitis, cholecystitis, and perforated peptic ulcer [15, 16, 17, 18]. In the present study, the reasons for conversion to open surgery were related to adhesions, with the exception of extensive irreversible intestinal ischemia; these factors could potentially cause iatrogenic bowel injury. Our study revealed that the number of previous laparotomies was significantly associated with conversion from laparoscopic to open surgery in patients with SSBO. Farinella et al. [19] reported a number of previous laparotomies ≤ 2 to be one of the predictors for successful laparoscopic adhesiolysis for adhesive small bowel obstruction. Additionally, Fu et al. [20] reported a history of multiple abdominal surgeries as one of the independent risk factors for recurrence of adhesive small bowel obstruction due to the inevitable formation of new abdominal adhesions caused by multiple operations. Thus, we consider that the increasing number of abdominal surgeries might lead to extensive and complex intra‐abdominal adhesions and that laparoscopic surgery for SSBO in patients with a previous history of multiple laparotomies should be performed with conversion to open surgery in mind. Additionally, a history of gastrointestinal surgery was also significantly associated with conversion in the univariate analysis. Gastrointestinal operations involve direct manipulation of the bowel and may produce dense and widespread adhesions extending beyond the upper abdominal cavity into the pelvis. These adhesions can impair visualization and limit safe laparoscopic manipulation, thereby increasing the likelihood of conversion. By contrast, obstetrical and gynecological surgeries generally result in adhesions confined to the pelvic cavity, which may have a lesser impact on small bowel handling during laparoscopic procedures. Thus, we believe that this anatomical difference may explain why gastrointestinal surgery history exhibited a stronger association with conversion to open surgery than gynecological procedures in our study. However, because the number of conversion events was small, these findings should be considered hypothesis‐generating rather than definitive, and no new or robust risk factors for conversion could be identified.

Our study had some limitations. First, this was a single‐center, retrospective study. Given the limited sample size, some comparisons may have been underpowered to detect statistically meaningful differences. Second, the surgical procedures and skin incision length were selected at the surgeon's discretion, thereby introducing potential selection bias and possible misclassification between laparoscopic and open approaches. Third, regional and organizational factors, including the lower BMI of the study population and irregular timing of emergency surgery, may have influenced the choice of surgical approach and could not be fully adjusted for in this retrospective analysis, representing potential sources of selection bias. Fourth, although propensity score matching was performed using objective preoperative variables such as inflammatory markers, severity scores, and CT findings as surrogate indicators of disease severity, residual confounding by indication could not be completely eliminated because the choice of surgical approach ultimately depended on the surgeon's clinical judgment. Fifth, long‐term outcomes such as recurrence, chronic abdominal pain, and quality of life were not evaluated owing to the retrospective design and limited availability of follow‐up data. These outcomes are clinically relevant and should be addressed in future prospective studies with structured long‐term follow‐up to better clarify long‐term outcomes. Therefore, prospective studies with larger sample sizes are needed to validate our results.

In conclusion, our study using PSM suggests that laparoscopic surgery for SSBO is beneficial for reducing the occurrence of postoperative complications. However, in patients with a history of multiple laparotomies or gastrointestinal surgery, laparoscopic surgery should be performed considering the possibility of conversion to open surgery.

## Author Contributions

**Toshimichi Kobayashi:** conceptualization, methodology, data curation, formal analysis, writing – original draft, visualization, project administration. **Ryota Suda:** investigation, writing – review and editing. **Hiroki Yamaguchi:** investigation, writing – review and editing. **Shoma Iida:** investigation, writing – review and editing. **Kanami Iwama:** investigation, writing – review and editing. **Takumi Seichi:** investigation, writing – review and editing. **Yoshihiro Nagae:** investigation, writing – review and editing. **Hiroyuki Higuchi:** investigation, writing – review and editing. **Akitoshi Ando:** investigation, writing – review and editing. **Itsuki Koganezawa:** investigation, writing – review and editing. **Masashi Nakagawa:** investigation, writing – review and editing. **Kei Yokozuka:** investigation, writing – review and editing. **Shigeto Ochiai:** investigation, writing – review and editing. **Takahiro Gunji:** investigation, writing – review and editing. **Toru Sano:** investigation, writing – review and editing. **Satoshi Tabuchi:** investigation, writing – review and editing. **Naokazu Chiba:** investigation, writing – review and editing. **Shigeyuki Kawachi:** conceptualization, methodology.

## Funding

The authors have nothing to report.

## Ethics Statement

This study was approved by the Ethics Committee of Tokyo Medical University (approval number T2019‐0169) and complied with the tenets of the Declaration of Helsinki.

## Consent

Informed consent for participation was obtained using an opt‐out form.

## Conflicts of Interest

The authors declare no conflicts of interest.

## Data Availability

The data that support the findings of this study are available from the corresponding author upon reasonable request.
